# Sacrospinous Hysteropexy—Video Tutorial of Construction and Application of a Feasible and Inexpensive Teaching Model for Simulation

**DOI:** 10.1007/s00192-024-05965-3

**Published:** 2024-11-05

**Authors:** Greta Lisa Carlin, Wolfgang Umek, Barbara Bodner-Adler, Fanny Carolina Mikula, Sören Lange

**Affiliations:** 1 Department of Obstetrics and Gynaecology, Medical University of Vienna, Waehringer Guertel 18–20, 1090 Vienna, Austria; 2 Department of Obstetrics and Gynaecology, University Hospital of Zurich, Zurich, Switzerland; 3 Institut für spezielle Gynäkologie und Geburtshilfe, Karl Landsteiner Institut, Vienna, Austria; 4 Medical University of Zurich, Zurich, Switzerland

**Keywords:** Sacrospinous hysteropexy, Surgical teaching model, Pelvic organ prolapse, Hystero-preserving operations

## Abstract

**Introduction and Hypothesis:**

Sacrospinous hysteropexy is one of the preeminent uterus-preserving surgical techniques for treating pelvic organ prolapse supported by level one evidence. As training on models greatly improves surgical skills and outcomes, we developed a simple and inexpensive model to simulate sacrospinous hysteropexy.

**Methods:**

A step-by-step instruction for the production of the model is available to be viewed online. To keep production costs low, readily available materials were used, with a total cost per model of about 2 EUR (Austria, August 2023). All important anatomical landmarks (prolapsing uterus, vagina, ischial spine and sacrospinous ligament) were all represented. We present a detailed instructional video on how to construct the model and the practical training, detailing the individual steps of a successful sacrospinous hysteropexy, available online. Thus, trainees are able to practice the individual movements of the entire surgical procedure on this simulator model guided by the tutorial video. In this way, trainees will be able to practice the entire surgical procedure.

**Results:**

An introduction to the model with explanation of all anatomical landmarks and a standardised explanation of the surgery with its individual steps (handout distributed).

**Conclusion:**

The presented video showcases the feasibility of the easy construction and application of a model for the surgical skill training of sacrospinous hysteropexy. Easily accessible, inexpensive material and its simple build make this a reproducible model regardless of geographic or socioeconomic resources.

**Supplementary Information:**

The online version contains supplementary material available at 10.1007/s00192-024-05965-3.

## Introduction

Pelvic organ prolapse is a bulging of the vaginal wall into, or out of, the vagina due to a weakness of the pelvis muscles and sagging of one or more pelvic organs. The lifetime risk is high, especially because of an increasingly aging population worldwide, and leads to a significant deterioration of the quality of life. Treatment options range from conservative to various surgical procedures performed laparoscopically, abdominally, or vaginally [1].

Sacrospinous hysteropexy (SSHP) has become a first-line treatment for pelvic organ prolapse based on level one evidence [2]. Compared with vaginal hysterectomy with uterosacral ligament suspension, SSHP was shown to have similar anatomical and subjective outcomes while having a shorter operating time, shorter hospitalisation, and lower intraoperative blood loss [3]. It is therefore an important urogynaecological operating technique for gynaecology trainees to master. However, learning vaginal operating techniques can be challenging, as visualisation of the operating steps performed by the surgeon is rather difficult for an observer, owing to the limited size of the vaginal canal as the primary operating site [4, 5]. Furthermore, surgical skills training using simulators and models has been shown to improve surgical performance, trainees’ confidence levels, and patients’ outcomes in different settings [6, 7]. To date, there have been no simulator models available for vaginal sacrospinous hysteropexy to the best of our knowledge.

## Materials and Methods

**Fig. 1:**
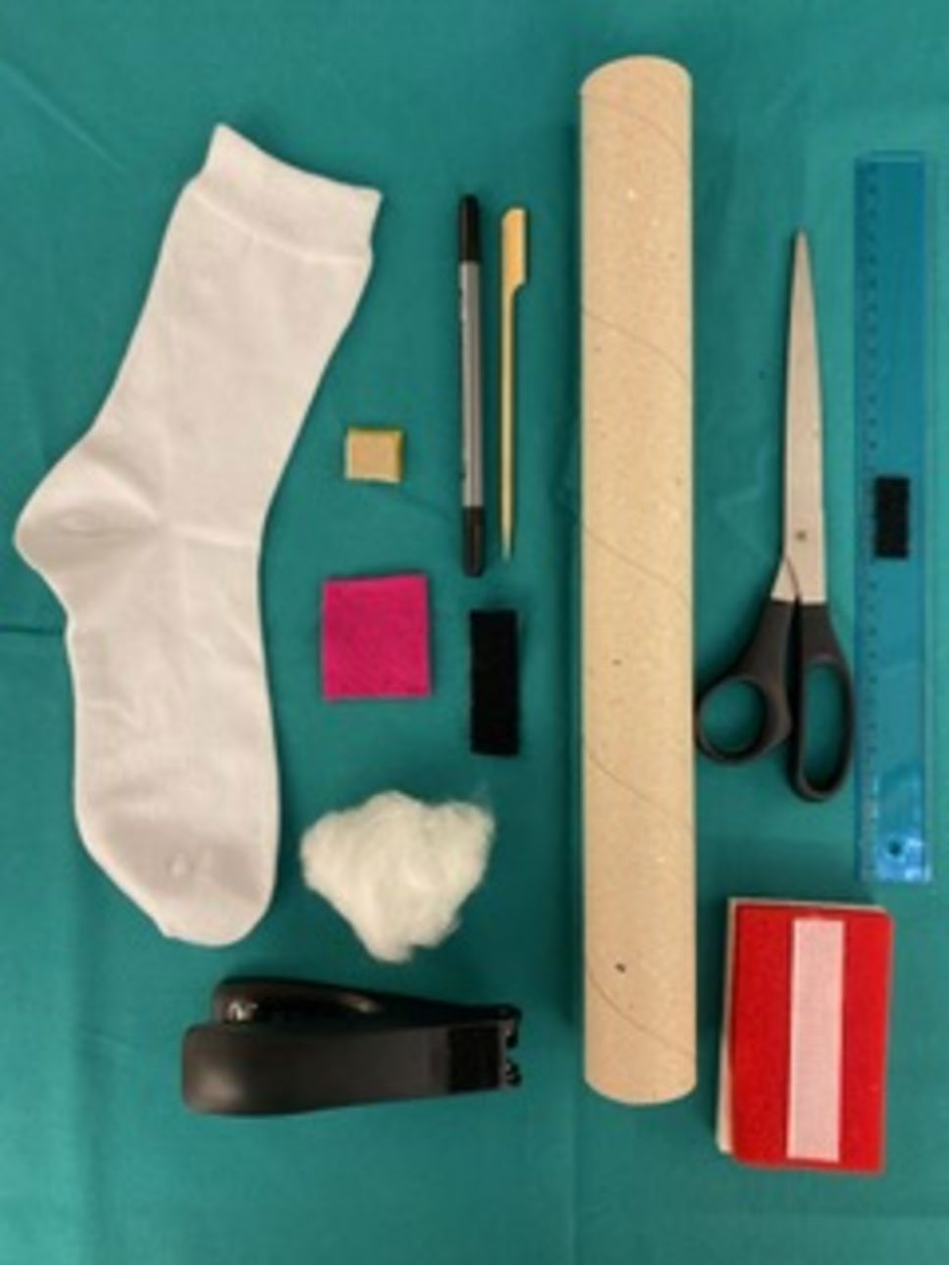
Required materials: sock, felt, felt patch, pen, skewer, hook and loop tape, stapler, paper roll, scissors, ruler, attachment site on a stable surface

**Fig. 2:**
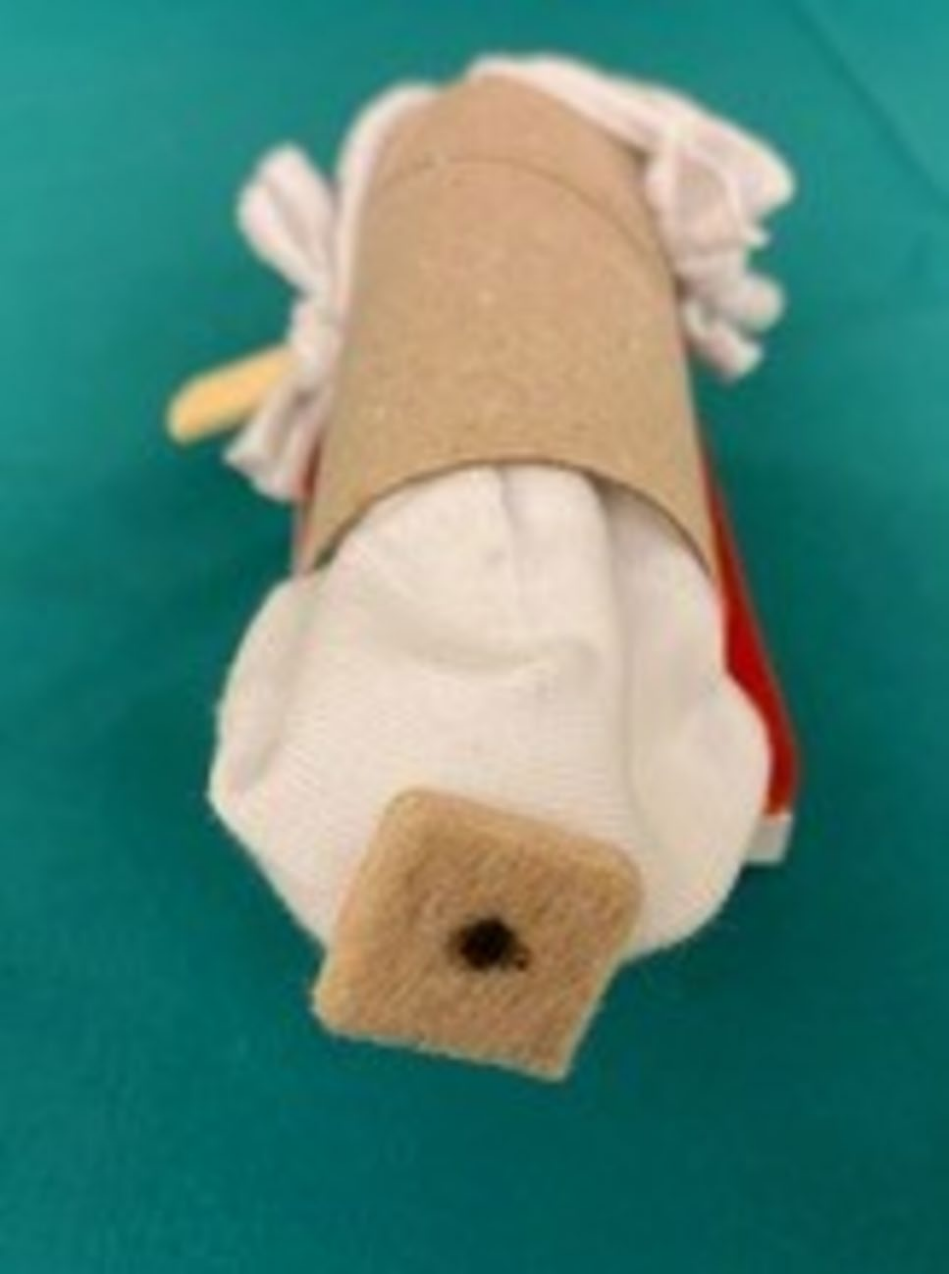
Finished model

We set out to construct a simple model to simulate sacrospinous hysteropexy from widely available, inexpensive and sustainable materials on which to easily practice the execution of the individual movements required during a vaginal sacrospinous hysteropexy in correct succession. The full construction of the model from its individual components is demonstrated step by step during the first part of the tutorial video (Supplemental digital content Appendix 1). Materials include a cardboard roll, a sock, loose cotton, felt, rubber stopper and a wooden skewer, all of which are readily available and inexpensive (Fig. 1). All anatomical landmarks relevant during sacrospinous hysteropexy (i.e. vagina, cervix, rectovaginal space, ischial spine and sacrospinous ligament) are represented in the model: the cardboard roll functions as the vaginal canal in which a cotton stuffed sock represents the vaginal wall and prolapsing uterus, attached to which there is a felt cervix. The sock’s loose cotton filling represents the rectovaginal space and a rubber stopper the ischial spine, adjacent to which a felt-covered wooden skewer functions as the sacrospinous ligament. Additionally, a piece of hook-and-loop tape was attached to the finished model so that it could be affixed to a stable surface, allowing for an easier training experience (Fig. 2). Total cost per model was calculated to be as low as 1.85 EUR (central Europe, September 2023), corresponding to about US$2. Construction time per model was measured to be 6 min and 30 s.

The second part of the video showcases a practical demonstration of the simulator model in use, detailing all necessary steps of a successful sacrospinous hysteropexy, as performed at our institution, demonstrated by an experienced urogynaecology surgeon. First, a high posterior colpotomy towards the posterior cervix is performed, followed by blunt preparation of the rectovaginal space to visualise the sacrospinous ligament. Then, a suture is placed through the ligament and thereafter through the posterior cervical wall, without being knotted yet. Next, the colpotomy is closed, and only afterwards is the pre-laid fixation suture tied, elevating and attaching the cervix towards the sacrospinous ligament.

## Results

**Fig. 3:**
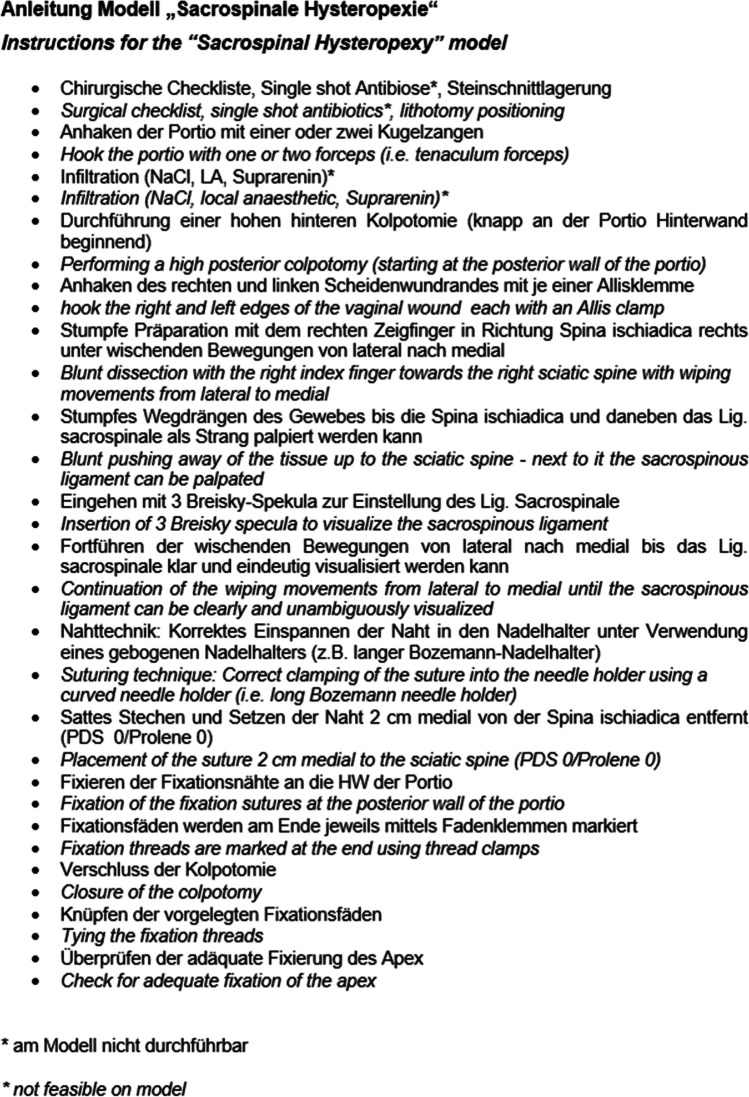
Handout detailing the surgical steps of sacrospinous hysteropexy in German, with English translation in cursive

The model was tested by 52 participants. All received an introduction to the model itself with an exact explanation of all anatomical landmarks. Additionally, a standardised explanation of the surgery’s individual steps was given and a handout detailing them in German (Fig. 3) distributed. After training all participants were asked for feedback by assessing the training they had received on a grading system from 1 to 5 (1 = excellent; 5 = very bad). The results were overwhelming positive.

## Discussion

This instructional video details the construction of a simple, realistic, inexpensive model for the surgical skills training of sacrospinous hysteropexy. It is designed to help increase a trainee’s operative performance by allowing them to practice the execution of each individual step of the surgical procedure. Feedback obtained from users after training was overwhelmingly positive.

## Supplementary Information

Below is the link to the electronic supplementary material.

Supplementary file1 (MP4 52012 KB)

## Data Availability

The datas generated and analyzed during this study are available from the corresponding author upon reasonable request.
